# Therapies for Cirrhotic Cardiomyopathy: Current Perspectives and Future Possibilities

**DOI:** 10.3390/ijms25115849

**Published:** 2024-05-28

**Authors:** Hongqun Liu, Daegon Ryu, Sangyoun Hwang, Samuel S. Lee

**Affiliations:** 1 Liver Unit, Cumming School of Medicine, University of Calgary, Calgary, AB T2N 4N1, Canada; hliu@ucalgary.ca (H.L.); gon22gon@naver.com (D.R.); mongmani@daum.net (S.H.); 2 Division of Gastroenterology, Yangsan Hospital, Pusan National University School of Medicine, Pusan 46033, Republic of Korea; 3 Department of Internal Medicine, Dongnam Institute of Radiological and Medical Sciences, Pusan 46033, Republic of Korea

**Keywords:** cirrhotic cardiomyopathy, treatments, beta blockers, antioxidants, anti-apoptosis, anti-inflammation

## Abstract

Cirrhotic cardiomyopathy (CCM) is defined as cardiac dysfunction associated with cirrhosis in the absence of pre-existing heart disease. CCM manifests as the enlargement of cardiac chambers, attenuated systolic and diastolic contractile responses to stress stimuli, and repolarization changes. CCM significantly contributes to mortality and morbidity in patients who undergo liver transplantation and contributes to the pathogenesis of hepatorenal syndrome/acute kidney injury. There is currently no specific treatment. The traditional management for non-cirrhotic cardiomyopathies, such as vasodilators or diuretics, is not applicable because an important feature of cirrhosis is decreased systemic vascular resistance; therefore, vasodilators further worsen the peripheral vasodilatation and hypotension. Long-term diuretic use may cause electrolyte imbalances and potentially renal injury. The heart of the cirrhotic patient is insensitive to cardiac glycosides. Therefore, these types of medications are not useful in patients with CCM. Exploring the therapeutic strategies of CCM is of the utmost importance. The present review summarizes the possible treatment of CCM. We detail the current status of non-selective beta-blockers (NSBBs) in the management of cirrhotic patients and discuss the controversies surrounding NSBBs in clinical practice. Other possible therapeutic agents include drugs with antioxidant, anti-inflammatory, and anti-apoptotic functions; such effects may have potential clinical application. These drugs currently are mainly based on animal studies and include statins, taurine, spermidine, galectin inhibitors, albumin, and direct antioxidants. We conclude with speculations on the future research directions in CCM treatment.

## 1. Introduction

Cirrhotic cardiomyopathy (CCM) is one of the most important complications in patients with cirrhosis. The definition includes systolic and/or diastolic dysfunction and morphological changes, such as chamber enlargement, without pre-existing heart disease. CCM was first termed in 1989 [1]. Since then, this entity has been investigated by many studies, which led to the first definition in 2005 at the World Congress of Gastroenterology in Montreal [2] called the WCG criteria. Following advances in imaging technology, the diagnostic criteria were redefined by a multidisciplinary expert group, resulting in the Cirrhotic Cardiomyopathy Consortium criteria (CCC criteria) [2]. The WCG criteria emphasize the blunted cardiac response to exercise, volume challenge, or pharmacological stimuli, whereas the CCC criteria are based on contractile function at rest (Table 1). Nevertheless, the newer CCC criteria appear superior [3,4] and should be used going forward.

Although CCM has been investigated extensively, the management is still not standardized because there is no clearly-accepted specific treatment. Due to the significant baseline peripheral vasodilatation in cirrhotic patients, vasodilators such as angiotensin-converting enzyme (ACE) inhibitors are unfeasible to treat CCM, as further vasodilation may lower the mean arterial pressure below the cutoff value (approximately 65 mmHg) that induces kidney injury. Patients with cirrhosis are not sensitive to cardiac glycosides; thus, these drugs cannot be used.

However, there are general supportive measures, current potentially useful therapies, and the future possibility of specific treatments for CCM, which will be summarized in the present review.

## 2. Clinical Relevance

CCM is clinically significant because when the cirrhotic heart is challenged, the subclinical dysfunction becomes overt. These challenges include exercise, transjugular intrahepatic portosystemic shunt (TIPS) insertion, drugs, and liver transplantation [5]. Regarding the last, during the transplantation procedure, intravenous fluids augment cardiac preload, and postoperatively, systemic vascular resistance is raised, which increases cardiac afterload. All these challenges significantly aggravate any pre-existing CCM. It was demonstrated that cardiovascular complications are the third-leading cause of death in patients after liver transplantation, accounting for 7–21% of deaths [6]. Even without any challenges, CCM plays an essential role in mortality. Premkumar and coworkers demonstrated that the mortality rates within 2 years of cirrhotic patients were parallel to the grades of left ventricular diastolic dysfunction (LVDD grade 1, 10.5% mortality; grade 2, 22.5%; and grade 3, 40%) [7]. Furthermore, LVDD also correlates with the incidence of acute kidney injury (OR 6.273, *p* < 0.05) and hepatic encephalopathy (OR 5.6, *p* < 0.05).

## 3. Pathogenic Mechanisms

Cirrhosis is defined as hepatic architectural damage characterized by nodular regeneration and diffuse fibrosis; these features lead to liver dysfunction and portal hypertension. Mechanisms underlying CCM and portal hypertension have been recently reviewed in detail [2]. Liver dysfunction impacts cardiac molecules, for example, decreased density of β-adrenergic receptors [8], an increased cholesterol-to-phospholipid ratio of the cardiomyocyte sarcolemmal plasma membrane [9], and abnormal contractile filaments, such as a myosin heavy chain (MHC) shift from the stronger α-MHC to the weaker and slower β-MHC isoform [10]. Portal hypertension causes intestinal vascular congestion, which results in bacterial translocation and endotoxemia. Under stimulation by lipopolysaccharides, pro-inflammatory cytokines, such as TNFα and interleukin (IL)-1β, are increased. These cytokines further augment nitric oxide and carbon monoxide, which inhibit cardiac contractility. Other cardiac contractile inhibitors include oxidative stress, apoptosis, and bile acids.

## 4. Management

There are currently no guidelines on the treatment of CCM. The general management of overt noncirrhotic heart failure usually requires oxygen and afterload and preload reduction [11]. Preload reduction includes water and sodium restriction and diuretics. Unfortunately, long-term diuretic application may cause electrolyte imbalances and renal injury [12]. Afterload reduction mainly consists of vasodilation. However, vasodilators are usually not suitable for treating heart dysfunction in cirrhosis because these patients often have significant vasodilatation and hypotension. Thus, there is a real risk that vasodilators may worsen a cirrhotic patient’s clinical condition [5]. Therefore, ACE inhibitors or angiotensin receptor blockers are not feasible in patients with advanced cirrhosis. The potential therapies for CCM may include nonselective beta-blockers (NSBBs. Table 2), antioxidants, and anti-apoptotic and anti-inflammatory agents.

### 4.1. Non-Selective Beta-Blockers

NSBBs have a long history in the therapy of cirrhotic patients. In 1980, Lebrec and colleagues [21] conducted a randomized clinical trial with propranolol, which demonstrated that it significantly decreased the portal pressure in patients with cirrhosis and portal hypertension, at doses reducing the heart rate by 25%. They speculated that propranolol might be valuable in preventing recurrent bleeding caused by esophageal varices based on the portal-hypotensive effect. Many subsequent studies have confirmed this initial speculation. NSBBs have thus been used in cirrhotic patients with portal hypertension for more than 40 years [21,22]. To the present date, NSBBs are still a standard of care to prevent variceal bleeding and rebleeding [23].

#### 4.1.1. Issues of NSBBs in Portal Hypertension

Since Lebrec and colleagues first explored the application of NSBBs in cirrhotic patients with portal hypertension [21], many studies have been conducted. Poynard et al. [13], in 1991, analyzed four randomized controlled trials. They concluded that patients with NSBBs not only had fewer first episodes of bleeding but also had improved survival rates. Almost 30 years later, Serste and colleagues, i.e., the Lebrec group [14], conducted a prospective study in 151 cirrhotic patients with refractory ascites. The patients were divided into two groups: one group received propranolol (n = 77), and the other group did not. They reported that the survival time in the propranolol group was shorter than that in the control group. Furthermore, the 1-year probability of survival was significantly lower in the propranolol group compared with controls. They concluded that NSBBs should not be used in cirrhotic patients with refractory ascites. However, a major problem of this study was that it was not randomized; patients were selected by their physician to receive NSBB or not, and thus, there was an inescapable likelihood of selection bias. Comparing the patients’ basic characteristics, in the propranolol group, the presence of esophageal varices and total bilirubin levels were significantly higher than in the controls, and the systolic blood pressure was significantly lower. These important differences of these baseline parameters strongly suggest that the patients in the propranolol group had more severe liver failure.

After further studies on the safety of NSBBs in advanced cirrhosis, a “window hypothesis” was proposed. The window hypothesis contends that NSBBs are neither useful nor necessary in the early stages of cirrhosis and potentially hazardous in the later stages, such as those patients with refractory ascites [24]. The sympathetic nervous system activity is nearly normal in the early stages of cirrhosis, and therefore, NSBBs will exert only modest effects at this stage; at the later end stage, although the sympathetic system is highly active, NSBBs at this stage not only inhibit the sympathetic system but also decrease the cardiac contractility and arterial pressure [24], which may result in tissue hypoperfusion and death. NSBBs may therefore only be clinically beneficial within a narrow ‘window period’ of the clinical course of cirrhosis.

However, recent studies do not seem to support the “window hypothesis”; Chen et al. [25] examined the National Health Insurance Research Database of Taiwan. Patients with cirrhosis taking propranolol vs. those not on this drug (controls) were matched for gender and age. The mean survival of cirrhotic patients with refractory ascites was 34.3 ± 31.2 months in the propranolol group and 20.8 ± 26.6 months in the control group (*p* < 0.001). They concluded that compared with controls, propranolol treatment reduces mortality. Leithead and coworkers [16] also demonstrated that even with refractory ascites, NSBB treatment confers benefits to cirrhotic patients with end-stage liver disease on the waiting list for liver transplantation.

Further evidence that demonstrated the beneficial effect of NSBBs is a study of patients with acute-on-chronic liver failure (ACLF). Mookerjee et al. [17] examined the effect of NSBBs on systemic inflammation in patients with ACLF and found that NSBBs significantly reduced white cell count and the concentration of plasma C-reactive protein (CRP). The severity of inflammatory reaction was an independent predictor for the development of ACLF after enrollment and for ACLF-associated mortality. NSBB treatment downregulated the grades of ACLF and decreased mortality rates. In contrast, patients without NSBB treatment tended to show a worsening of ACLF during their hospital stay. Moreover, patients who discontinued NSBB treatment had significantly higher 28-day and 3-month mortality rates [17].

An important caveat to emphasize is that all the above studies and indeed all previous comparative studies of NSBBs and mortality in advanced cirrhosis are nonrandomized and thus inevitably suffer from probable selection bias. This limitation decreases the strength of any conclusions that can be drawn from these studies. Nevertheless, at present, the tentative conclusion based on the most recent evidence suggests that in cirrhotic patients, NSBBs should be stopped only if and when the mean arterial pressure drops below 65 mmHg, as that is the approximate cut-off value at which renal hypoperfusion occurs [26].

#### 4.1.2. NSBBs for CCM Treatment

There are two pathways by which NSBBs theoretically could exert a therapeutic effect on CCM. One pathway is by blocking direct cardiac damage due to an overactivated sympathetic nervous system. The other is the beneficial effect of the decrease in portal hypertension.

The pathway of direct cardiac damage of the overactivated sympathetic nervous system is unrelated to portal hypertension. In patients with cirrhosis, the sympathetic nervous system is overactivated, manifesting as persistent adrenergic activation and high circulating levels of catecholamines [27]. The heart is one of the target organs that can be damaged by high levels of circulating catecholamines. An animal study demonstrated that an increase in portal and hepatic sinusoidal pressure leads to the activation of sympathetic nerves to the heart [28]. It is well known that sympathetic overactivation plays an important role in a variety of pathophysiological processes in cardiovascular diseases [29]. Cao et al. [30] furthermore specified that it is the β-adrenergic receptor (β-AR) overactivation that is a major pathological factor mediating cardiac inflammatory injury and causing cardiac dysfunction. Cardiac inflammatory injury is a key mechanism underlying the development of cardiac diseases [31].

Regarding the portal hypertension-related pathway, in cirrhotic patients, the portal venous hypertension causes mesenteric congestion. The congested gut impairs bowel motility and consequently leads to increased intestinal permeability and bacterial overgrowth [32,33]. The bacterial overgrowth stimulates the production of endotoxin, and the increased intestinal permeability augments the absorption of endotoxin. Moreover the dysfunctional cirrhotic liver has reduced detoxication capability, and the presence of portosystemic collateral circulation enables endotoxin to directly enter the systemic circulation. All the above changes in patients with cirrhosis cause endotoxemia and systemic inflammation, a phenomenon termed the ‘inflammatory phenotype’. In summary, an inflammatory phenotype seems to underlie disease severity in many cirrhosis-related complications, including CCM.

Portal hypertension-associated inflammation is an essential pathogenic event in CCM. NSBBs are demonstrated to decrease portal pressure; thus, this class of drugs theoretically could have a therapeutic effect on CCM. Another mechanism by which NSBBs could decrease endotoxemia is by increasing bowel motility and reducing intestinal permeability, thus decreasing bacterial translocation [34]. NSBBs have anti-inflammatory effects, which may be beneficial in CCM because this condition displays an inflammatory phenotype: inflammatory cytokines, such as TNFα and IL-1β, are increased in the cirrhotic heart. Furthermore, NSBBs improve both systolic and diastolic function in patients with non-cirrhotic chronic heart failure [35]. However, there is no solid evidence to date to demonstrate that NSBBs have clear therapeutic effects on CCM.

Because of the observations above, many centers have investigated the possible therapeutic effect of NSBBs on CCM. Although current theories suggest that NSBBs may exert therapeutic effects, the pertinent studies have not confirmed this.

Although theoretically it is rational to use NSBBs to treat CCM, there are some difficulties in the clinical application. First, although cardiac function at resting status is normal, i.e., left ventricular ejection fraction (LVEF) is preserved due to the vasodilatation, the contractile responsiveness is decreased, such as decreased global longitudinal train (GLS, <18%). Furthermore, diastolic function is also abnormal, manifested as a reduced relaxation velocity of ventricular muscle (diastolic mitral annular velocity for example). Unfortunately, NSBBs possess not only anti-inflammatory effects but also inhibit the contractility-stimulating β_1_-AR, therefore potentially further inhibiting cardiac systolic and diastolic function.

In a recent randomized controlled trial, Premkumar et al. [18] enrolled 189 cirrhotic patients divided into 3 groups: carvedilol (an alpha- and beta-blocker) alone, carvedilol + ivabradine (a cardiac pacemaker current [*I_f_*] blocker), and standard medical therapy (SMT) for 52 weeks. The targeted heart rate reduction (THR) was defined as heart rate reduction to 55–65 beats per minute. They observed that patients treated with carvedilol + ivabradine showed an improvement of LVDD and improved survival compared with the SMT group. Even the patients treated with carvedilol alone showed modest improvements in cardiac and clinical parameters. In patients who obtained THR with carvedilol treatment, the E/e’ was insignificantly decreased by 0.6%. In comparison, there was a 14.2% increase in E/e’ in the SMT group (0.6% vs. 14.2%, *p* = 0.003). These data confirmed a therapeutic effect of carvedilol on diastolic dysfunction. One issue to mention is that this study did not specifically report the therapeutic effect of carvedilol on cirrhotic patients with refractory ascites and Child–Pugh class C. The most promising results with the combination carvedilol + ivabradine therapy are encouraging and warrant further study in larger trials.

Silvestre and colleagues [15] performed a randomized, double-blinded, placebo-controlled trial to evaluate the effect of 6 months of metoprolol on CCM, randomizing 41 patients to the metoprolol group and 37 to a placebo group. Thirty-eight patients in the metoprolol group and thirty-five in the placebo group finished the study. The study did not show any significant differences in the improvement of stroke volume or diastolic dysfunction. Indeed, no echocardiography parameter or morphology was significantly different between the metoprolol and placebo groups. Furthermore, metoprolol treatment did not change the levels of noradrenaline, plasma renin activity, and troponin compared with the placebo group. Clinical events, such as hospitalization and mortality rates, were not different significantly between the two groups. Therefore, the authors concluded that six months of metoprolol treatment does not improve cardiac function and morphology in patients with CCM. However, randomization may have produced selection bias by chance: 19.5% of patients in the metoprolol group were Child–Pugh class C, whereas this percentage in the placebo group was only 8.1%. Thus, the different severity of cirrhosis in the two groups may have contributed to a type II error.

Another prospective study consecutively enrolled 403 patients, 213 with compensated cirrhosis and 190 with decompensated cirrhosis [36]. This study reported that NSBBs were more effective on the heart and less effective on portal pressure in patients with decompensated cirrhosis than in those with compensated cirrhosis. At baseline, decompensated patients were more hyperdynamic than compensated patients, with higher heart rate and cardiac output (CO), lower arterial pressure, and higher portal pressure. NSBBs had greater reductions in heart rate (15 ± 12 vs. 10 ± 11, *p* < 0.05) and CO (17 ± 15% vs. 10 ± 21%; *p* < 0.01) in decompensated patients. However, NSBBs induced less portal pressure decrease in decompensated patients than in compensated patients (10 ± 18% vs. 15 ± 12%; *p* < 0.05). Furthermore, the CO decrease was an independent predictor of mortality in decompensated patients: compared with survivors, NSBBs produced a greater decrease in CO in decompensated patients who died (21 ± 14% vs. 15 ± 16%; *p* < 0.05). Death risk was higher in decompensated patients with CO < 5 L/min than in those with CO > 5 L/min. Based on the data above, these authors concluded that NSBBs may be detrimental in patients with end-stage cirrhosis and latent cardiomyopathy because NSBBs further reduce the cardiac compensatory reserve.

A potential benefit is that NSBBs shorten the prolonged QTc interval and decrease the risk of ventricular arrhythmias [37,38]. There is no controversy regarding this effect.

Why are the NSBB study results so discrepant? Does a therapeutic window also exist in CCM treatment with NSBBs? The explanations may be due to patient and NSBB selection. Some patients might have disease progression during the time course of treatment, such as those with ACLF. Moreover, several other variables, such as the patients’ nutritional status, and differences in other standard medical therapies may also play a role.

In terms of NSBB selection, several different drugs have been used in studies. These include propranolol, nadolol, and carvedilol, all of which are true NSBBs, exerting effects on both β_1_ and β_2_ receptors. In addition, carvedilol is also an α_1_-adrenergic blocker. On the other hand, metoprolol, also studied in CCM, is a selective β_1_-receptor blocker. All these differences may contribute to the observed discrepant therapeutic effects in patients with CCM.

### 4.2. Potential Therapies in CCM (Table 3)

**Figure 1:**
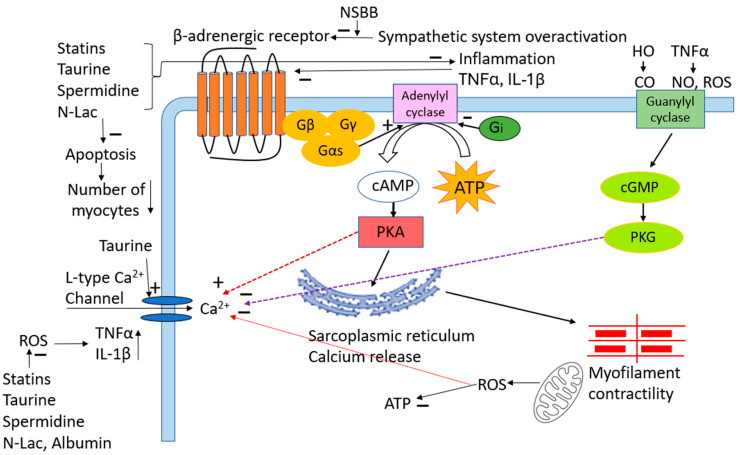
Schematic diagram of pathogenic mechanisms of CCM and therapeutic targets. + denotes positive or stimulatory effect. —denotes negative or inhibitory effect. NSBB: non-specific beta-blocker. TNFα: tumor necrosis factor alpha. IL-1β: interleukin 1beta. HO: hemo oxygenase. CO: carbon monoxide. NO: nitric oxide. ROS: reactive oxygen species. PKA: protein kinase A. PKG: protein kinase G. N-Lac: N-acetyllactosamine.

There is currently no accepted specific treatment for CCM. As detailed above, NSBB therapy is controversial. Other potential strategies could be suggested by the pathogenesis of CCM, such as antioxidants and anti-inflammatory and anti-apoptotic substances (Figure 1). The study by Taprantzia et al. [39] reported that compared with healthy controls, oxidative indicators, such as lipid peroxidation and malondialdehyde (MDA) levels, were significantly increased in cirrhotic patients, thus showing that oxidative stress is significantly augmented in cirrhosis [40]. Our previous studies demonstrated that cardiac inflammation, oxidative stress, and apoptosis play a significant pathogenic role in CCM [41,42,43]. Agents active against oxidative stress, inflammation, and apoptosis may therefore have potential in clinical application to treat CCM.

**Table 3:** Potential therapies in cirrhotic cardiomyopathy.

First Author (Ref.)	Substance	Mechanism of Action	Species/Model	Effects
Bortoluzzi [44]	Albumin	Decreases inflammatory and oxidative stress	CCl_4_-cirrhotic rats	Enhances systolic function
Fernandez [45]	Albumin	Reduces systemic inflammation	Patients with decompensated cirrhosis	Improves cardiac function
Mousavi [46]	Taurine	Reduces oxidative stress, protein carbonylation, improves mitochondrial function, and increases ATP levels	Bile duct-ligated cirrhotic rats	protects liver and heart from injury
Sheibani [47]	Spermidine	Decreases inflammatory and oxidative stress	Bile duct-ligated cirrhotic rats	Enhances systolic function, decreases QTc
Yoon [48]	Galectin-3 inhibitor (*N*-acetyllactosamine)	Decreases inflammation by inhibiting TNFα	Bile duct-ligated cirrhotic rats	Increases blood pressure; enhanced systolic and diastolic function
Niaz [49]	Statin (atorvastatin)	Decreases inflammation and oxidative stress	Bile duct-ligated cirrhotic rats	Increases chronotropic response to isoproterenol; decreases QTc interval.
Node [50]	Statin (simvastatin)	Attenuates systemic inflammation	Patients with dilated cardiomyopathy	Improves LVEF, NYHA classification

CCl_4_: carbon tetrachloride; QTc: corrected QT interval; TNFα: tumor necrosis factor alpha; LVEF: left ventricular ejection fraction; NYHA: New York Heart Association functional classification.

#### 4.2.1. Statins

Statins not only inhibit cholesterol synthesis and downregulate the serum cholesterol level but also possess antioxidant and anti-inflammatory effects. Bielecka-Dabrowa et al. [51] investigated the role of atorvastatin on the parameters of inflammation and left ventricular function in patients with dilated cardiomyopathy (DCM). They showed that atorvastatin significantly reduced the inflammatory cytokines in plasma, such as TNFα and IL-6. It also decreased the cardiac dysfunction marker N-terminal pro-brain natriuretic peptide (BNP) concentration. Atorvastatin significantly improved cardiac function as manifested by decreased left ventricular diastolic and systolic diameters. Furthermore, it significantly increased LVEF. Finally, this drug also significantly increased the probability of 5-year survival.

Niaz et al. [49] tested the effects of atorvastatin on cirrhotic hearts induced by bile duct ligation (BDL) in rats. They reported that the chronotropic responses of atria from BDL rats to isoproterenol were decreased compared with those from sham-operated controls. The response was increased in BDL rats treated with atorvastatin. Furthermore, the QTc interval and serum BNP, TNFα, and MDA levels were increased in BDL rats, and atorvastatin significantly decreased these parameters. In summary, atorvastatin improved the chronotropic hyporesponsiveness and downregulated the oxidative stress and inflammation in cirrhotic rats. From the evidence above, both in humans and animal models, statins seem to exert a therapeutic effect on cardiac function, mediated via the inhibition of inflammation, apoptosis [52], and oxidative stress. Therefore, statins are potentially useful therapeutic agents that need further study. Moreover, given its already proven excellent safety profile, it can safely be used in almost all patients with cirrhosis except perhaps those with severely decompensated liver function.

#### 4.2.2. Taurine

Taurine possesses multiple functions, including the modulation of protein phosphorylation, calcium ion regulation, membrane stabilization, bile acid conjugation, lipid metabolism, glucose regulation, antioxidation, anti-inflammation, and anti-apoptosis [53,54]. It is an abundant amino-sulfonic acid in many tissues, such as skeletal muscle, liver, platelets, and leukocytes, especially in electrically excitable tissues, such as the heart [54]. Low taurine serum levels have been closely associated with many oxidative stress-mediated pathologies, including hepatic disorders and cardiomyopathy [55]. It plays a significant role in reducing lipid peroxidation products [54], therefore protecting cells from tissue damage [56]. It also exerts a protective effect on oxidative stress-induced vascular dysfunction [57], which may also apply to the heart [55].

Pion et al. [58], in a feline model, showed that taurine depletion leads to cardiomyopathy. Another study [59], using a taurine transporter-knockout model in mice, showed that these animals naturally develop cardiac dysfunction. Beyranvand and coworkers [60] verified that taurine supplementation increases the exercise capacity in patients with heart failure, and this effect is partially due to the antioxidant role of taurine.

All these data suggest that taurine is essential for cardiovascular function. However, the role of taurine in CCM is not well studied. Since the biosynthesis of taurine is primarily in the liver [54], cirrhosis decreases the functional liver mass and consequently the synthesis of taurine [5].

It is known that the cardiac content of taurine is significantly decreased in the cirrhotic heart [61] and parallel to the decrease in taurine is the decreased antioxidant capacity in these hearts. Thus, the supplementation of taurine may be potentially beneficial. Taurine has been shown to reduce lipid peroxidation and protect cells from damage [56]. Liu and coworkers [62] created a model of transverse aortic constriction-induced heart failure in mice and demonstrated that taurine exerts a protective effect on cardiac function. The mechanisms are due to a decrease in myocyte oxidative stress, apoptosis, hypertrophy, and cardiac fibrosis.

The results obtained from non-cirrhotic heart failure may also be applicable to CCM. In the BDL rat model of cirrhosis, Mousavi et al. [46] showed that oxidative stress, including lipid peroxidation, reactive oxygen species, protein carbonylation, and the GSH/GSSG ratio, were significantly increased in the cirrhotic heart. Taurine administration significantly reduced tissue oxidative stress and increased the total antioxidant capacity and mitochondrial ATP content [46].

In summary, taurine decreased oxidative stress and improved mitochondrial function in the cirrhotic rat heart. Moreover, it also reduced creatine kinase MB (CK-MB), a surrogate marker of heart injury. Taurine is therefore a valuable candidate treatment that warrants further human studies in CCM.

#### 4.2.3. Spermidine

Spermidine, like taurine, also possesses antioxidant, anti-inflammatory, and anti-apoptotic effects [47,63]. Chen et al. [64] used a transverse aortic constriction model in mice to investigate the role of spermidine in heart failure (HF). They divided the animals into four groups: sham controls, HF, HF + spermidine, and HF + spermidine antagonist (trans-4-methylcyclohexylamine (4-MCHA)). They reported that spermidine significantly decreased the left ventricular mass. The most significant changes in echocardiographic parameters were in the HF mice treated with 4-MCHA. This group demonstrated further increases in left ventricular systolic and diastolic diameters, left ventricular end-diastolic, and diastolic volumes and further decreases in LVEF. Moreover, 4-MCHA significantly increased the cardiac content of galectin-3, an inhibitor of cardiac function [48]. Finally, the mice treated with 4-MCHA showed the greatest extent of cardiomyocyte apoptosis. These data demonstrated that spermidine inhibition worsened cardiac function and spermidine improved cardiac function in heart failure.

Omar et al. [65] evaluated the impact of spermidine in a rat model of acute myocardial infarction (AMI) induced by isoproterenol. Compared to the untreated group, spermidine significantly reversed electrocardiographic RR interval, QRS, QT intervals, and ST segments towards normal ranges. Furthermore, serum CK-MB and lactate dehydrogenase, the parameters of cardiac injury, were significantly reduced by spermidine. Furthermore, compared with the untreated AMI group, spermidine significantly rescued the reduced antioxidant capacity [65]. Martinalli et al. [66] administered spermidine to patients with peripheral artery disease and reported that it increased maximal walking distance and reduced oxidative stress in these patients. What is the effect of spermidine on CCM? Sheibani et al. [47] investigated the effects of spermidine in the BDL-induced cirrhotic rat. They showed that it significantly decreased the QTc interval, which is consistent with a study of Omar and colleagues [65].

Furthermore, compared with the control group, spermidine significantly reduced the cardiac oxidative stress and inflammation: decreased levels of malondialdehyde, increased superoxide dismutase and GSH, and decreased TNFα and IL-1β. Moreover, the contractility of isolated ventricular papillary muscles from the BDL + spermidine group was significantly increased compared with BDL controls. These studies give us hope that spermidine may one day be applicable to cirrhotic patients with cardiovascular dysfunction.

#### 4.2.4. Galectin-3 Inhibitor

Galectins are members of the lectin family. Galectin-3 is one of the 15 mammalian galectins identified to date [67]. Galectin-3 is widely distributed in the nucleus, cytoplasm, cell surface, extracellular space, and the blood circulation [68]. It is closely associated with CCM because (1) galectin-3 levels are significantly increased in cirrhotic patients [69] and animal models of liver fibrosis [70]. Moreover, galectin-3 is increased in the cirrhotic heart [48]. (2) It serves pleiotropic functions, including inflammation [71], oxidative stress, and apoptosis [72], which are pathogenic mechanisms of CCM. Galectin inhibitors therefore are theoretically attractive to investigate for CCM treatment.

We [48] investigated the role of galectin-3 in CCM pathogenesis, using N-acetyllactosamine (N-Lac) as a galectin-3 inhibitor. We divided rats into four groups, sham operated controls, sham + N-Lac, BDL, and BDL + N-Lac. In these animals, the left ventricular content of galectin-3, pro-inflammatory cytokine TNFα, BNP, the collagen I and III ratio, blood pressure, and cardiomyocyte contractility were measured. We demonstrated that galectin-3, TNFα, BNP, and the collagen I and III ratio were significantly increased in the hearts isolated from BDL rats compared with those from sham controls. Blood pressure and systolic and diastolic contractile velocities were significantly decreased in cardiomyocytes isolated from BDL rats. The galectin-3 inhibitor significantly decreased levels of galectin-3, TNFα, BNP, and the collagen I/III ratio in cirrhotic hearts and significantly increased the blood pressure and improved the cardiomyocyte contractile velocities of the BDL rats. N-Lac had no effect on sham controls. The galectin-3 inhibitor decreased the cardiac content of TNFα and improved the depressed contractility in the cirrhotic heart. With the data above, we concluded that the increase in galectin-3 in the cirrhotic heart plays an important role in the inhibition of cardiac contractility. This effect is mediated via TNFα.

#### 4.2.5. Albumin

Albumin is synthesized exclusively by the liver, so its serum levels are reduced in acute and/or chronic liver disease [73]. It may be a candidate for the treatment of CCM for the following reasons: (1) Hypoalbuminemia is common in patients with advanced cirrhosis. Thus, improving hypoalbuminemia should reduce ascites formation by increasing plasma colloid osmotic pressure. (2) Albumin decreases the protein expressions of Gαi_2_, TNFα, and iNOS [44], which are known inhibitors of cardiac contractility. Albumin decreases TNFα via 2 mechanisms, binding serum TNFα and blunting the overexpression of TNFα in cardiac tissue. (3) It decreases oxidative stress [74], which is an important initiator of inflammation. Albumin binds many substances, such as NO, reactive oxygen species (ROS), and proinflammatory cytokines, which may be involved in the pathogenesis of both peripheral arterial vasodilatation and cardiac dysfunction in patients with cirrhosis. (4) Albumin increases adenylate cyclase 3, the enzyme that catalyzes ATP to cAMP [44], and is thus a key mediator of the ventricular-stimulatory pathway.

Bortulozzi and coworkers [44] used carbon tetrachloride (CCl_4_) to induce cirrhosis and ascites in rats, subsequently infusing intravenous albumin to determine its effects on the cirrhotic heart. They demonstrated that the cardiac expression of TNFα, iNOS, and NAD(P)H-oxidase activity were significantly increased in the cirrhotic heart, and cardiac contractility was significantly decreased in cirrhotic rats compared to controls. Albumin infusion reversed the protein expressions of TNFα, iNOS, and NAD(P)H-oxidase to control levels, and the depressed cardiac contractility also reversed back to normal.

A clinical study also demonstrated the role of albumin in cardiac contractility in patients with cirrhosis. The Pilot-PRECIOSA study [45] demonstrated that patients who received a high albumin dose (1.5 g/kg weekly) showed improvement in systolic function with increases in cardiac index and left ventricular stroke work index.

Because the antioxidant and volume-expanding properties of albumin, regardless of any possible cardioprotective effects, are beneficial in almost all patients with cirrhosis and it lacks any significant downside, we believe this therapeutic agent is highly promising and could be considered at any stage of cirrhosis, not just those with advanced disease.

#### 4.2.6. Direct Antioxidants

Hydrogen is a direct antioxidant. The small size of the hydrogen molecule allows it to easily penetrate the cell membrane to the cytosol. It is naturally metabolized without residue, and therefore, there are no side effects [75]. Similar to taurine and spermidine, hydrogen has antioxidant [76], anti-inflammatory, and anti-apoptotic effects [77,78]. Jing et al. [79] tested the effect of hydrogen-rich saline on isoproterenol-induced myocardial infarction (MI) in rats. They reported that hydrogen-rich saline decreased MDA, increased superoxide dismutase, and decreased serum TNFα and IL-6 in the MI heart. Furthermore, hydrogen-rich saline decreased cardiac CK-MB levels in the MI rats compared to control rats. Hydrogen-rich saline pretreatment also reduced the infarct size, alleviated pathological changes in the left ventricle, and improved cardiac function.

Lee et al. [80] tested the effect of hydrogen on BDL-induced cirrhosis in rats, finding that hydrogen-rich saline significantly decreased thiobarbituric acid-reacting substances (TBARS) and MDA, markers of oxidative stress, and increased superoxide dismutase and GSH, which are markers of antioxidant reserves in BDL rats. Consistent with the study of Jing et al. [79], hydrogen-rich saline reduced pro-inflammatory markers, including TNFα, IL-1β, and IL-6. The study of Lee and colleagues did not test the role of hydrogen-rich saline on direct cardiac function. Instead, they showed an improvement of hyperdynamic circulation. Qian et al. [81] used hydrogen-rich water (4 mL/kg orally three times a day) to treat patients with chronic graft-versus-host disease and demonstrated that it prolonged the survival time and increased the survival rate during 4 years of treatment. They speculated that these therapeutic effects were mediated via the antioxidant and anti-inflammatory effects of hydrogen. Accordingly, we suggest that hydrogen may improve cardiac function in CCM because hydrogen-rich saline attenuates oxidative stress and inflammation in subjects with cirrhosis, and these phenomena are pathogenic mechanisms of CCM.

### 4.3. Liver Transplantation

Liver transplantation remains the definitive ‘cure’ for cardiovascular anomalies of cirrhosis. A recent study showed that within one year after liver transplantation, 34% of CCM patients recovered according to the 2005 Montreal criteria and 57% according to the 2019 CCC criteria [82]. However, the recovery process is challenging, and the overall cardiovascular system experiences both risks and benefits. As stated above, the benefits accrue over a longer term, whereas many of the risks occur during the perioperative and short-term postoperative states. During the procedure of liver transplantation, the hemorrhage and clamping of the major blood vessels may cause hypovolemia, whereas aggressive fluid resuscitation may cause volume overload. Perioperative hemodynamic fluctuations significantly affect cardiac function. Other factors, such as acidosis, hypothermia, and electrolyte disturbances, may impair cardiac contractility [83]. Citrated blood transfusion may cause hypocalcemia [84], which further depresses cardiac contractility.

After liver transplantation, the peripheral vascular resistance immediately increases, as does the blood pressure, which raises both cardiac preload and afterload. These challenges may result in overt cardiac failure in patients with CCM [85]. Another challenge of liver transplantation is the shortage of donor organs, which limits its application. The high cost, the complexity of the procedure, the need for the long-term use of immunosuppressants, and complications such as infections and rejection also limit its widespread clinical application. In many economically underdeveloped global regions, liver transplantation is simply not available.

## 5. Future Possibilities

Since the traditional therapeutic strategies for non-cirrhotic cardiac dysfunction, such as vasodilators, are not applicable in CCM, other potential treatments have been investigated over the past decade. In particular, therapies aimed at correcting the pathogenesis-related targets, such as antioxidants and anti-inflammatory and anti-apoptotic agents, may be beneficial to patients with CCM. To date, these strategies are mostly limited to animal research, so these agents need to be validated in well-designed clinical trials.

Another therapeutic potential agent is NSBBs. Theoretically, NSBBs should have therapeutic effects on CCM. However, the results from different studies are inconsistent. Currently, NSBBs are only a standard of care for the prevention of primary and secondary bleeding caused by gastroesophageal varices. Borrowing from the therapeutic concept of systemic hypertension, which needs lifelong treatment, we may also need to treat portal hypertension lifelong rather than just administering NSBBs when variceal bleeding forces us to do so. Because portal hypertension is an important pathogenic factor underlying CCM, treating portal hypertension may lead to the improvement of CCM.

## Figures and Tables

**Table 1:** Diagnostic criteria systems for cirrhotic cardiomyopathy.

Criteria	Systolic Dysfunction	Diastolic Dysfunction
WCG criteria (2005)	LVEF < 55% Or Blunted increase in contractility on stress testing	E/A ratio < 1.0 Or DT > 200 ms Or IVRT > 80 ms
CCC criteria (2019)	LVEF ≤ 50% Or GLS < 18%	≥3 of the followings E/e’ ratio ≥ 15 e’ septal < 7 cm/s TR velocity > 2.8 m/s LAVI > 34 mL/m^2^

WCG: World Congress of Gastroenterology; CC: cirrhotic cardiomyopathy consortium; LVEF: left ventricular ejection fraction; E/A: E-wave to A-wave ratio; DT: mitral deceleration time; IVRT: isovolumetric relaxation time; GLS: global longitudinal strain, absolute value; TR: tricuspid regurgitation; LAVI: left atrial volume index.

**Table 2:** Effects of beta-blockers.

First Author (Ref.)	NSBB	Subjects	Effects
Poynard [13]	Propranolol, nadolol	Patients	Decreases bleeding, improves survival
Sersté [14]	Propranolol	Patients with refractory ascites	Decreases 1-year survival rate
Silvestre [15]	metoprolol	Patients with CCM	No change in stroke volume or diastolic function
Leithead [16]	Propranolol, carvedilol	Patients with refractory ascites	Improves survival
Mookerjee [17]	Propranolol	Patients with ACLF	Improves inflammation and survival
Premkumar [18]	carvedilol + ivabradine	Patients with CCM	Improves LVDD and survival
Zambruni [19]	Nadolol	Patients with cirrhosis	Decreases QTc in patients with prolonged QTc over 1–3 months
Henrikson [20]	Propranolol	Patients with cirrhosis	Decreases QTc over 90 min

NSBB: non-specific beta-blocker. ACLF: acute-on-chronic liver failure. CCM: cirrhotic cardiomyopathy. LVDD: left ventricular diastolic dysfunction. QTc: corrected QT interval.

## Data Availability

Not applicable.
